# Quantum Entanglement in Double Quantum Systems and Jaynes-Cummings Model

**DOI:** 10.1186/s11671-017-1985-0

**Published:** 2017-03-31

**Authors:** Paweł Jakubczyk, Klaudiusz Majchrowski, Igor Tralle

**Affiliations:** Faculty of Mathematics and Natural Sciences, University of Rzeszów, Pigonia Str. 1, 35–310 Rzeszów, Poland

**Keywords:** Ballistic electron transport, Double quantum well structure, Qubit, Nonclassical light, Jaynes-Cummings model

## Abstract

In the paper, we proposed a new approach to producing the qubits in electron transport in low-dimensional structures such as double quantum wells or double quantum wires (DQW). The qubit could arise as a result of quantum entanglement of two specific states of electrons in DQW structure. These two specific states are the symmetric and antisymmetric (with respect to inversion symmetry) states arising due to tunneling across the structure, while entanglement could be produced and controlled by means of the source of nonclassical light. We examined the possibility to produce quantum entanglement in the framework of Jaynes-Cummings model and have shown that at least in principle, the entanglement can be achieved due to series of “revivals” and “collapses” in the population inversion due to the interaction of a quantized single-mode EM field with a two-level system.

## Background

The field of research termed as Quantum Information Theory and, more specifically, Quantum Computation attracts nowadays a great deal of attention. It is not easy or rather impossible to make a review even the most important and authoritative publications devoted to these issues in a short Introduction. However, to mention just a few, here are [1, 2] and the immense number of papers cited therein. For our purposes, it is sufficient to point out an important text by David Di Vincenzo [3] in which he formulated the so-called Di Vincenzo’s check list, the list of requirements the quantum system has to fit in, for one has the possibility to implement on such a basis the quantum computer, the Holy Grail for those who deal with quantum information and quantum computation. These requirements are the following: (i) well-defined qubits; (ii) relatively long decoherence times; and (iii) initial state preparation and some others equally important; we do not however concentrate on them right now.

It is worth mentioning that there were many attempts to build scalable quantum computer in terms of spin qubits; see for instance [4–9].

Not long ago, it was proposed [10, 11] that the electron ballistic transport can be used for these purposes, since many if not all of the requirements from the Di Vincenzo’s list could be met in this way. The main idea of the papers [10, 11] is to use for that purpose double quantum wire structure to mimic the qubit. The authors of these publications envisaged that by proper adjusting the structure parameters, the system can be designed in such a way as to produce, due to the electron tunneling across the structure, an assigned transfer of the electron wave function between two wires, while the ballistic electrons move along the wires. The physical qubit would then consist of two adjacent quantum wires, while the logical state |0〉 would be defined by the presence of a single electron in one of them and the logical state |1〉 by the presence of the electron in an another. It is worth mentioning that similar coherent electron oscillations which occur in double quantum wire structure were theoretically predicted many years ago, while the experimental observation of this effect is still dubious [12].

Our aim here is, following the general scheme proposed in [11], to advance somewhat different approach, in which qubit would arise as a result of quantum entanglement of two specific states of electrons in double quantum well or double quantum wire (DQW) structure. These two specific states are the symmetric and anti-symmetric (with respect to inversion symmetry) states arising due to tunneling across the structure, while entanglement could be produced and controlled by means of the source of nonclassical light. The paper is organized as follows. At first, we briefly discuss the model which we use, then discuss the possible physical realization of proposed scheme for achieving the quantum entanglement in DQW structures.

## Methods

We start from the observation that in accordance with von Neumann conjecture (see [13]), all observables are equally accessible for manipulation with quantum states. Thus, in many realizations of a generalized 2-state system (generalized spin $\hat {\sigma }_{z}$), there is a “natural” choice of such states [14]. Hilbert space corresponding to such generalized spin is two-dimensional, and the eigenvectors making its basis are chosen to be the eigenvectors of the $\hat {\sigma }_{z}$-operator sometimes denoted as ∣*↑*〉 and ∣*↓*〉 with the eigenvalues ±1, so that

$$\hat{\sigma}_{z}\mid\uparrow\rangle=\mid\uparrow\rangle,\quad \hat{\sigma}_{z}\mid\downarrow\rangle = -\mid\downarrow\rangle. $$

For example, in case of electrons moving between two potential wells separated by a barrier, the “natural” basis identifies the eigenstates of *σ*_*z*_ with the ground states |*L*〉 and |*R*〉 in the separate wells, left and right, and just this basis was chosen in the Refs. [10, 11]. There is, however, another possibility. It is well known that due to tunneling, the states in two quantum wells which are close to each other and separated only by thin barrier are split into two. In the work [15], it was shown experimentally and theoretically that these states, arising due to tunneling in double quantum well (DQW) structure and which are termed as symmetric ∣*Ψ*_*S*_〉 and anti-symmetric ∣*Ψ*_*A*_〉 with respect to coordinate inversion, sustain their individuality during electron ballistic transport within such structure. It means, neither electron-electron interaction nor electron-phonon interaction, that is interaction with “environment”, lead to their mixing. So, we assume that in DQW system the exact integral of motion to exist, and it can be written in the form (see for example [16]):

$$\hat{P}=\sigma_{z}\hat{S}=\sigma_{z}\exp (i\pi a^{\dagger}a),\quad \sigma_{z} = \left(\begin{array}{cc} 1 & 0\\ 0 & -1 \end{array}\right), $$

where *a* ^*†*^ and *a* are the fermionic creation and annihilation operators. Thus, the exact state vector of the system depends on two quantum numbers and is the solution of the following equations:

$$\hat{H}\mid\Psi_{np}\rangle = \epsilon_{np}\mid\Psi_{np}\rangle, \quad \hat{P}\mid\Psi_{np}\rangle = p_{np}\mid\Psi_{np}\rangle, $$

where the numbers *p*=±1 define the parity and *n*=0,1,2… are the energy quantum numbers for the steady-state eigenvalues *ε*_*np*_. If we suppose for simplicity that the single quantum well has only one quantum state (which however, as we shall see, does not impact on the generality of our consideration), then in the DQW-system composed of two similar QW, there is the splitting of quantum level into two due to tunneling between QWs. One of them is termed symmetric while another one as anti-symmetric with respect to space coordinate inversion. In this case, the Hamiltonian of two-level system can be written as

$$\hat{H}_{0}=\frac{1}{2}\hbar\omega\sigma_{z}, $$

where $\hbar \omega = \Delta _{SAS}$ and *Δ*_*SAS*_ is the splitting between symmetric and anti-symmetric states; these two states make “pseudo-spin” states, and the spinor $\left (\begin {array}{c}1\\ 0\end {array}\right)$corresponds to the state ∣*Ψ*_*as*_〉, $\hat {P}\mid \Psi _{as}\rangle =-\mid \Psi _{as}\rangle $ while the spinor $\left (\begin {array}{c}0\\ -1\end {array}\right)$ to the state $\hat {P}\mid \Psi _{s}\rangle =+\mid \Psi _{s}\rangle $. We do not take into account the electron’s spin degree of freedom, because there is no external magnetic field and Zeeman splitting.

The interaction between the two-level system and the quantized electromagnetic field (EM) in dipole approximation is still the same as in the semi-classical case:

$$\hat{V}=-e\vec{r}\cdot\vec{E}=-\hat{d}\hat{E}, $$

where $\hat {d}$ is the dipole moment operator and $\hat {E}$ becomes the electric field operator. For the single-mode EM field we have

$$\hat{H}_{F}=\hbar\Omega\left(b^{\dagger}b+1/2\right) $$

and the interaction operator

$$\hat{V}=\hbar\left(b+b^{\dagger}\right)\left(g\sigma_{+}+g^{*}\sigma_{-}\right), $$

where *b* ^*†*^ and *b* are the bosonic creation and annihilation operators; $\sigma _{+}=\left (\begin {array}{cc}0&1\\ 0&0\end {array}\right)$ and $\sigma _{-}=\left (\begin {array}{cc}0&0\\ 1&0\end {array}\right)$are the spin-flip matrices, and $g=\left (\wp \varepsilon _{\Omega }/2\hbar \right)\sin (kz)$ is the electric dipole matrix element. Here, *Ω* is the single-mode field oscillation frequency, *k*=*Ω*/*c* is the wave number, *ε*_*Ω*_ is the “electric field per photon,” and the squiggle *℘* is the component of dipole moment along $\vec {E}$ (see Ref. [17]). With the two-level Hamiltonian $\hat {H}_{0}$, free field Hamiltonian *H*_*F*_, and interaction operator, the total DQW field Hamiltonian becomes:

$$\hat{H}=\frac{1}{2}\hbar\omega\sigma_{z}+\hbar\Omega b^{\dagger}b +\hbar\left(b+b^{\dagger}\right)\left(g\sigma_{+}+g^{*}\sigma_{-}\right). $$

Since the spin-flip operators have the Heisenberg-type time dependence [17], using rotating wave approximation, we get a bit simpler form of DQW field Hamiltonian:

$$\hat{H}=\frac{1}{2}\hbar\omega\sigma_{z}+\hbar\Omega b^{\dagger}b+\hbar g\left(b\sigma_{+}+b^{\dagger}\sigma_{-}\right), $$

which is nothing else but the Hamiltonian of Jaynes-Cummings model (JCM), one of the most important models of*Quantum Optics* [18]. This model, despite its conceptual simplicity, exhibits interesting and nontrivial features. Namely, it turns out that Rabi oscillations, which are the common feature of two-level “atom” interacting with *classical* EM field, are damped *independently of the number of photons*, a result that sometimes called “Cummings collapse” (see Ref. [17, 18] for details). However, for longer times, the system exhibits a series of “revivals” and “collapses,” which in our opinion, could be used to make the electrons in DQW structure to be entangled. The consequences of such revivals arising when one uses the source of nonclassical light is discussed in the next paragraph.

In order to have the qubits, we need first of all to have the Hilbert space of lowest possible dimensionality. For that purpose, one has to have the possibility to inject the single electrons one by one into DQW structure, which initially is empty, that is, which does not contain the free electrons. In the paper [11], it was suggested that the DQW structure can be produced on base of *GaAs*. The authors assumed the donor concentration in GaAs to be equal to 10^13^cm^−3^ and remarked that at the temperature of 1 K, the electron concentration in conduction band is about 10^−5^cm^−3^, which gives in principle the possibility to achieve this goal, that is, to have a structure which is empty at the initial stage. First of all, it should be noted that to get the impurity concentration at the level of 10^13^cm^−3^ in *GaAs* is by no means a trivial task; rather, it is on the very edge of nowadays technology. The second objection is that the temperature of about 1 K is perhaps not very interesting from the practical point of view. We agree, however, that the general scheme proposed in [11] is worth to be treated seriously. Thus, we propose to use the DQW structure based on *Si*−*Ge*/*Ge* compound, because to purify *Si* and *Ge* up to 10^11^cm^−3^ or even 10^10^cm^−3^ is possible [19, 20].

### Calculations of Electron Concentration

Here, we briefly describe the calculations of electron concentration in Si conduction band versus *N*_*d*_ and the temperature. Suppose that we have the donor impurities of only a single type with the concentration *N*_*d*_. Then, taking into account the neutrality condition $n = p + N^{+}_{d}$, where *n*, *p*, and $N^{+}_{d}$ are the concentrations of electrons, holes, and ionized impurities, and assuming *p*≪*n*, it can be shown (see, for example [21]) that

1 $$ N_{c}\Phi_{1/2}(\eta)=N_{d}\left[ 1+g_{d}\exp\left(\frac{E_{F}-E_{d}}{k_{B}T}\right)\right]^{-1},   $$

where $N_{c}=2\left (\frac {2\pi m^{*}k_{B}T}{(2\pi \hbar)^{2}}\right)^{3/2}$ is the conduction band density of states, *m* ^∗^—electron effective mass and *k*_*B*_– Boltzmann constant, and *Φ*_1/2_ is 1/2 - Fermi integral, and $\eta = \frac {E_{F}-E_{c}}{k_{B}T}$. Here, *E*_*F*_ and *E*_*c*_ are the Fermi energy and the energy corresponding to the bottom of the conduction band; *g*_*d*_ and *E*_*d*_ are the degeneracy and ionization energy of a donor impurity, respectively. In our calculations, we assume *g*_*d*_=1, *E*_*c*_−*E*_*d*_=0.01 eV, and *N*_*d*_ ranging from 10^10^to 10^11^cm^−3^. This equation can be solved numerically by iteration procedure. It can be also observed that at first step, one can use the following approximation for the Fermi integral:

$$\Phi_{1/2}\approx \exp(\eta)(1+0.27\exp(\eta))^{-1}, $$

which gives the value of *Φ*_1/2_ with the accuracy not worse than 3*%* for *η*≤1.3. This approximation is valid at the very low impurity concentration and very low temperature. Then, using this approximation, we can at first iteration step calculate the Fermi energy, solving the corresponding equation. It gives

$$\begin{aligned} E_{F}&=E_{d}+k_{B}T \ln\left[\left(\frac{1}{4}\left(1-0.27\frac{N_{d}}{N_{c}}\right)^{2}+\frac{N_{d}}{N_{c}}\right.\right.\\ &\left.\left.\qquad\exp\left(\frac{E_{c}-E_{d}}{k_{B}T}\right) \right)^{1/2} - \frac{1}{2}\left(1-0.27\frac{N_{d}}{N_{c}}\right)\right]. \end{aligned} $$

At the second step, we used the following approximation for *Φ*_*j*_ (remember, in our case $j=\frac {1}{2}$), obtained in [22]:

$${} \Phi_{j}(\eta)=\left\{ \exp(-\eta)+\frac{\Gamma(j+2)2^{j+1}}{\left[ b+ \eta +\left(|\eta -b|^{c} + a^{c}\right)^{1/c} \right]^{j+1}} \right\}, $$

which gives the value of Fermi integral with the accuracy not worse than 1.2*%* for all values of *η* and $-\frac {1}{2}<j<4$. The results of calculations of the electron concentration in Si-conduction band in accordance with these formulae are shown in Fig. 2 as the surface in the coordinate frame: “donors concentration *N*_*d*_” and the “temperature *T*”.

## Results and Discussion

**Fig. 1:**
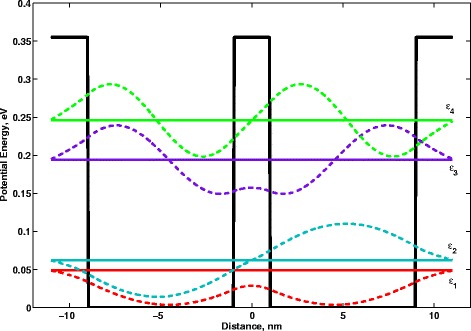
The energy spectrum of *Si*_0.16_ *Ge*_0.84_/*Ge* double quantum well and the corresponding stationary wave functions (*colored dashed curves*)

In Fig. 1 and Table 1, we present the calculations related to the DQW structure considered in the paper. The proposed structure can be produced on the basis of *Si*_1−*x*_ *Ge*_*x*_/*Ge* heterostructure [23], where two Si wells are separated by the *Si*_0.16_ *Ge*_0.84_ barrier of about 0.35 eV height. The following values of parameters were used in simulations: $m^{*}_{well}= 0.041 m_{e}$, $m^{*}_{bar}=0.0594 m_{e}$ (here $m^{*}_{well}$ and $m^{*}_{bar}$ (see Ref. [23]) are the electron effective masses in the well and in the barrier, respectively, and *m*_*e*_ is the free electron mass). In the simulations, different values of the well (*a*) and barrier (*b*) widths were used; they are presented in Table 1. In this table, *ε*_1_ and *ε*_2_ correspond to the energies of two quantum levels arising due to splitting of the ground states caused by the electron tunneling across the barrier; *Δ*_*SAS*_ is the splitting, *U*_0_ is the height of the barrier, while *ω* is nothing else but $\omega =\Delta _{SAS}/\hbar $. In Fig. 1, we depicted the DQW structure which is composed of two quantum wells of 8-nm width each and the barrier of 2-nm width. In this case, there are four quantum levels arising due to tunneling across the barrier: *ε*_1_≈0.0552 eV, *ε*_2_≈0.0734 eV, *ε*_3_≈0.227 eV and *ε*_4_≈0.286 eV. The corresponding splittings are equal: $\Delta ^{'}_{SAS}=0.0182$ eV and $\Delta ^{''}_{SAS}=0.0591$ eV, and the corresponding frequencies are *ω*_*SAS*1_=2.7711×10^13^ Hz and *ω*_*SAS*2_=8.98×10^13^ Hz. The simulations were made by means of *Piece-Wise Constant Potential Barriers Tool* [24]. In order to carry out them, one should determine the widths of the successive layers of which the DQW structure is composed, the values of electron effective mass within each layer as well as the dependence of potential on the space coordinate. The energy spectrum of DQW structure and the wave functions were calculated with the use of *Transfer Matrix Method* [25].

**Table 1:** Parameters of QW used in calculations

	*a*, nm	*b*, nm	*U*_0_, eV	*ε*_1_, eV	*ε*_2_, eV	*Δ*_*SAS*_, eV	*ω*×10^13^, Hz
1	4	2	0.355	0.1188	0.1765	0.0577	8.7624
2	5	3	0.355	0.1039	0.1264	0.0225	3.4169
3	5	4	0.355	0.1087	0.1209	0.0122	1.8494
4	6	4	0.355	0.0779	0.1088	0.0309	4.6838

**Fig. 2:**
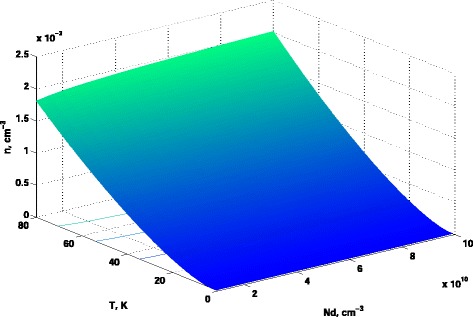
Electron concentration in Si conduction band vs donor concentration *N*_*d*_ and the temperature

The results of electron concentration calculations in the conduction band of *Si*, on base of which the DQW structure could be constructed, are presented in Fig. 2. From Fig. 2, one can easily notice that the electron concentration in the conduction band is *n*≪1 up to the temperature of 80 K. It is worth mentioning that the additional advantage of DQW structure is that the dipole moment which appears in the interaction Hamiltonian above, in case of DQW structure, is huge in comparison with that of atoms or molecules.

**Fig. 3:**
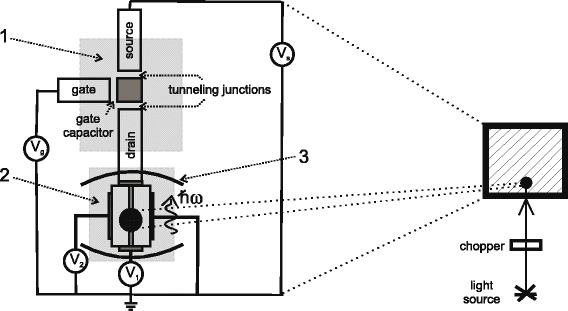
Layout of possible experiment: *1*—SET, *2*—DQW (see text), and *3*—microcavity

Then, similar to [11], in order to inject the electrons into DQW structure one by one, we propose to use the single-electron transistor (SET). The possible layout of the structure is depicted in Fig. 3. The second important ingredient of our model is the nonclassical light incident on the structure composed of DQW structure plugged into quantum or microcavity (see Fig. 3) and the SET. The microcavity is necessary in order to exclude the losses of photons; to the best of authors’ knowledge, the revivals were observed experimentally only in microcavities [26]. As one can easily see in Fig. 1, the voltage *V*_2_ is applied across the structure, while the voltage *V*_*s*_ is along it. The first one is supposed to be very small and needed to fulfill the boundary conditions: the electron incident on the barrier from, say, the left and not from the right. The voltage applied along the structure enforces the electrons to move in the plane perpendicular to the QW growth direction (in case of double quantum well structure) or along the wires (in case of double quantum wire structure).

Thus, in such structure, one can get the bi-partite pure states entanglement; indeed, in our case, two subsystems are the electron (subsystem A) which can be either in the state ∣*Ψ*_*AS*_〉 or in the state ∣*Ψ*_*S*_〉 and the photons (subsystem B). The state ∣*Ψ*〉∈*H*_*A*_⊗*H*_*B*_ is the product state if there exist ∣*ϕ*_1,2_〉_*A,B*_∈*H*_*A,B*_ such that ∣*Ψ*〉=∣*ϕ*_1_,*ϕ*_2_〉; otherwise, the state is called entangled [27]. The product states are ∣0〉⊗∣*n*〉, where ∣0〉 is associated with symmetric electron state and ∣*n*〉 with the EM field state characterized by the number of photons *n*, and ∣1〉⊗∣*n*−1〉, where ∣1〉 is associated with anti-symmetric one and ∣*n*−1〉 with the EM field state characterized by the number of photons *n*−1, whereas one of the entangled states in our case is

$$\mid \Psi_{ent} \rangle\equiv \left(\beta \mid 0\rangle \otimes \mid n\rangle + \gamma\mid 1\rangle\otimes\mid n-1\rangle \right), $$

where |*β*|^2^+|*γ*|^2^=1.

The product states ∣0〉⊗∣*n*〉 and ∣1〉⊗∣*n*−1〉 are referred to as “bare” states of Jaynes-Cummings model. As long as the electron transport in DQW structure is ballistic and the symmetric and antisymmetric states maintain their individuality (which we assume they do; as we already mentioned, in [15], it was shown that neither electron-electron interaction nor electron-phonon interaction mix them), we can treat these states as pure. It is worth mentioning that any pure state can be considered as superposed state, since it is such in every possible basis except that one, for which it is itself one of the basis states. As we shall see, it plays some role in the subsequent discussion.

It is well-known that in order to create entangled states out of product states, one needs to have interaction. In our case, it is nothing else but the interaction of electron in DQW structure with quantized EM field (photons). In accordance with what was said above, consider more general pure state of two-level system under the action of quantized EM field and suppose the electron injected into DQW structure initially is in the superposition of the states ∣*Ψ*_*s*_〉 and ∣*Ψ*_*as*_〉: ∣*Ψ*(0)〉_*electron*_=*C*_*s*_∣*Ψ*_*s*_〉+*C*_*as*_∣*Ψ*_*as*_〉. It can be achieved by means of SET injecting electrons of the certain energy into DQW structure. We can also assume the EM field is initially in the state $\mid \Psi (0) \rangle _{field} = \sum _{n=0}^{\infty } C_{n} \mid n\rangle $. Thus, the solution of corresponding Schrödinger equation with the Hamiltonian above is (see, for example, [18]:

2 $$ {\begin{aligned} \mid \Psi(t) \rangle &= \sum_{n=0}^{\infty} \left(\left[ C_{a} C_{n} \cos(gt \sqrt{n+1}) \right.\right.\\ &\quad \left.\left.-i C_{s} C_{n+1} \sin(gt \sqrt{n+1}) \right] \mid \Psi_{a} \rangle +\right. \\ &\quad+ \left[\!-i C_{a} C_{n_{1}} \!\sin(gt \sqrt{n}) +\! C_{s} C_{n} \cos(gt \sqrt{n}) \right] \!\mid \Psi_{s} \rangle \left.\!\right)\! \mid n \rangle. \end{aligned}}  $$

This state as it is clearly seen, in general, is entangled.

As we already mentioned, the most striking and interesting phenomena related to JCM are the “Cummings collapse” of Rabi oscillations and a series of revivals and collapses in the population inversion due to the interaction of a quantized single-mode EM field with a two-level system. The revival property discovered in [28, 29] is much more unambiguous signature of quantum electrodynamics than the collapse, since it is entirely due to the “grainy nature” of photon field.

The dynamics of Jaynes-Cummings model can be described also in terms of “dressed states”; using the basis of these states and taking into account that the detuning (see below) whatever small, usually is not equal to zero, it is convenient to describe the dynamics of JMC in terms of probability of the two-level system to be excited or in terms of “population inversion” (see [17, 28–30]) as

3 $$ W(t)=\sum_{n=0}^{\infty} \frac{|\alpha|^{2n} e^{-|\alpha|^{2}}}{n!}\left(\frac{\Delta^{2}}{\Omega_{n}^{2}} + \frac{4g^{2}(n+1)}{\Omega_{n}^{2}}\cos(\Omega_{n}t\right),   $$

where *W*(*t*) is the population inversion at *t*>0, $\Omega _{n}=\sqrt {\Delta ^{2}+4g^{2}(n+1)}$ is the Rabi frequency, and *Δ* is the detuning which in our case is equal to $\Delta =\Delta _{SAS}-\hbar \Omega $; we assume it to be *Δ*≥0; *g* is the strength of interaction between the two-level system and radiation field, which is also assumed to be positive. Another important parameter characterizing the model is |*α*|^2^, which is nothing else but the initial number of photons at time *t*=0, before the interaction between two subsystems (two-level “atom” and the photons) starts. The sum (3) is the series whose terms are of alternating signs. It has been calculated approximately in [28, 29] by means of replacing it by some integral; the integral was subsequently calculated by the saddle point method. In [30], it was pointed out that in this approximation, the estimates of reminder term have not been done and that is why the authors of [30] proposed another method for their evaluation, based entirely on *Number Theory* techniques. The last approach allows to get the analytic formulae which are as precise as possible in the sense that they allow to establish the limits of their possible applicability. In particular, the method elaborated in [30] and the final asymptotic formula derived there work well only if the number of photons in the incident flux *m*=|*α*|^2^≥100. It is definitely too large a number for the light source to be considered as nonclassical.

So, since in our case we are dealing with nonclassical light, we should capitalize on some different method for estimating the sum (3). It is not difficult to prove that despite the fact the system behavior depends strongly on the parameters which describe the model, the series (3) *converges absolutely and uniformly for any* |*α*|^2^, *Δ*, and *g*. Indeed, first of all, it is clear that

$$ \sum_{n=0}^{\infty}\frac{|\alpha|^{2n}e^{-|\alpha|^{2}}}{n!}=1. $$

One can write the sum (3) in terms of the two others, just like how it has been done in [30]: *W*(*t*)=*W*_1_+*W*_2_(*t*), where

$$W_{1}=\sum_{n=0}^{\infty}\frac{|\alpha|^{2n}e^{-|\alpha|^{2}}}{n!} \cdot \frac{4g^{2}(n+1)}{\Delta^{2}+4g^{2}(n+1)}, $$

$$W_{2}(t)=\sum_{n=0}^{\infty}\frac{|\alpha|^{2n}e^{-|\alpha|^{2}}}{n!} \cdot \frac{4g^{2}(n+1)}{\Delta^{2}+4g^{2}(n+1)}\cos(\Omega_{n}t). $$

Then, one has the following obvious chain of inequalities:

$$\begin{aligned} &\sum_{n=0}^{\infty}\frac{|\alpha|^{2n}e^{-|\alpha|^{2}}}{n!} \cdot \frac{4g^{2}(n+1)}{\Delta^{2}+4g^{2}(n+1)}\cos(\Omega_{n}t)\\ &\le \sum_{n=0}^{\infty}\frac{|\alpha|^{2n}e^{-|\alpha|^{2}}}{n!} \cdot \frac{4g^{2}(n+1)}{\Delta^{2}+4g^{2}(n+1)} \\ &\le \sum_{n=0}^{\infty}\frac{|\alpha|^{2n}e^{-|\alpha|^{2}}}{n!}. \end{aligned} $$

**Fig. 4:**
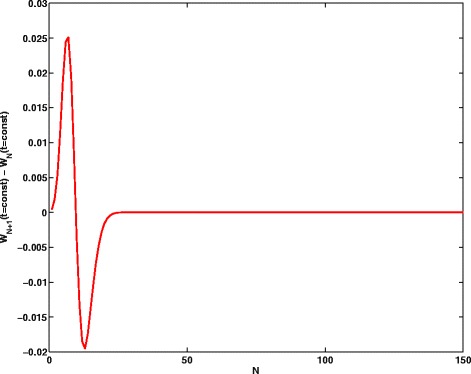
Population inversion difference *W*_*N*+1_(*t*=*const*)−*W*_*N*_(*t*=*const*) vs upper boundary *N* in the sum (3)

**Fig. 5:**
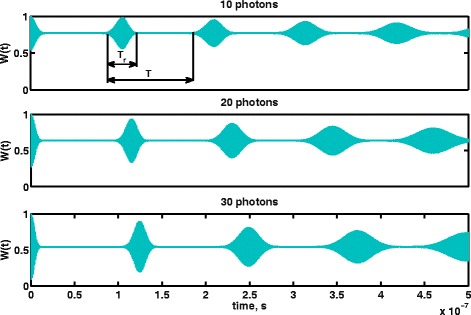
Revivals and collapses in population inversion in two-level system interacting with quantized EM field. In the calculations, we used the following values of parameters: *Δ*=*ω*−*Ω*=5×10^9^ *Hz*, *ω*=5×10^12^ *Hz*, *g*=4.15×10^8^ (CGS)

Now, it is obvious that the series (3) converges absolutely and uniformly at any |*α*|^2^ and any positive *Δ* and *g* by virtue of *Weierstrass Majorant Theorem*. The only question is how quickly the series (3) converges. Keeping this question in mind, let us take a pragmatic approach and just check it by *brute force* using the direct computer simulations; the results are plotted in Fig. 4. In this way, we estimated the upper boundary *N* for the sum (3), when (*N*+1)-sum and *N*-sum differ less than, say, 10^−13^; it turns out that *N*=41 for some value of *t*. Taking *N*=150, we calculate the population inversion *W*(*t*) for the two-level system. The results of calculations are depicted in Fig. 5 where one can clearly see the number of revivals and collapses.

One can also clearly see that the sum terms are adding together “interfering constructively” for some periods of time, while for the other periods of time, it looks like as if they cancel each other out. Each term oscillates at a particular Rabi frequency $\Omega _{n} = \sqrt {\Delta ^{2} + 4g(n+1)}$, and if two neiboring terms are oscillating out of phase with each other, say, with phase difference equal about *π*, they cancel each other out. If the neiboring terms are more or less in phase with each other, they interfere constructively.

The sum *W*(*t*) is aperiodic function of time, but for the first few revivals, the time lapses corresponding to the collapses are almost the same. In order to carry out quantum computations, we should, at first, adjust the clock frequency of hypothetical quantum computer to the “frequency” *f*=1/*T* of series of revivals and collapses. Since the function (3) strictly speaking is not periodic, the frequency *f* and the period *T* can be considered as well defined only for the first few revivals and collapses. Second, the time of calculations must be shorter than the time *T*_*r*_ (the time of single revival), and the information transmission has to be done during a few first quasi-periods of revivals and collapses.

We can add also as general remark that in our opinion, the coherent oscillations during the revivals of Rabi oscillations play the role which would have been played by coherent oscillations between two parallel quantum wires considered in the Refs. [10, 11]. As we already mentioned, the last ones were not definitely observed in the experiments, while the revivals were [26]. In our opinion, it is because in spite of ballistic transport in DQW structures, environmental decoherence, caused, for example, by the scattering of electrons by the edges of the structure and which cannot be eliminated, leads to the destruction of electron wavefunctions’ phase relations and hence to the loss of coherence. In order to avoid misunderstanding, we would like to emphasize that despite the fact that we started our discussion (see “Background” section) with mentioning Refs [10, 11], in which the electrons are assumed to move ballistically in the considered structures, we do not suppose them to do so in our case, because in our structure, they interact with the environment and strictly speaking their transport is not ballistic. However, *sensu stricto* ballistic transport is not necessary in our case, because as it is already mentioned, the symmetric and antisymmetric states are not mixed by the electron-electron and electron-phonon interaction. What is equally important, is that the quantum entanglement occurs due to electron interaction with external quantized EM field. This interaction is stronger than interaction with the environment and that is why the coherent oscillations survive and were observed in the experiments with microcavities.

## Conclusion

The aim of our work is to advance a new approach to producing the qubits in electron ballistic transport in low-dimensional structures such as double quantum wells or double quantum wires (DQW). The qubit would arise as a result of quantum entanglement of two specific states of electrons in DQW structure. These two specific states are the symmetric and antisymmetric (with respect to inversion symmetry) states arising due to tunneling across the structure, while entanglement could be produced and controlled by means of the source of nonclassical light. We examined the possibility to produce quantum entanglement in the framework of JCM and have shown that at least in principle, the entanglement can be achieved due to some interesting phenomena related to JCM, namely series of revivals and collapses in the interaction of a quantized single-mode EM field with a two-level system. The other characteristic feature of the entanglement which we propose to get in the ballistic transport in the DQW structure is that in accordance with our calculations, it can be achieved at relatively high temperature of about 80 K. To our mind, the second advantage of the proposed approach is that one could construct the quantum register by means of single DQW, since the number of quantum levels and hence, the number of symmetric and antisymmetric states and in the consequence, the number of qubits depend only on the depths of QWs and their geometric characteristics. The initial number of quantum levels in a single QW (when the tunneling does not occur) cannot be made of course too great; it is restricted by the available semiconductor materials, and it is very unlikely to be greater than four or five. Nevertheless, in this way, one can get a small quantum register by means of only the single DQW structure. Above all, using SET, it is possible to inject the electrons not only one by one but also in pairs. The consequences of this could be interesting, although this one as well as the possibility to make the universal quantum gates on such a basis require further analysis. Finally, we would like to mention interesting papers [31, 32], in which the electron quantum ballistic transport through a short conduction channel as well as the role of Coulomb interaction in modifying the energy levels of two-electron states in such channel were investigated. It seems interesting to find the relations between the findings of Refs [31, 32] and the possibility to produce quantum entanglement and qubits in the structures similar to ours. This however also requires more thorough analysis and could be the task for future research.
